# Feasibility of using respiration‐averaged MR images for attenuation correction of cardiac PET/MR imaging

**DOI:** 10.1120/jacmp.v16i4.5194

**Published:** 2015-07-08

**Authors:** Hua Ai, Tinsu Pan

**Affiliations:** ^1^ Department of Imaging Physics The University of Texas MD Anderson Cancer Center Houston TX; ^2^ Graduate School of Biomedical Sciences The University of Texas Health Science Center at Houston Houston TX USA

**Keywords:** PET/MR, cardiac, attenuation correction, motion

## Abstract

Cardiac imaging is a promising application for combined PET/MR imaging. However, current MR imaging protocols for whole‐body attenuation correction can produce spatial mismatch between PET and MR‐derived attenuation data owing to a disparity between the two modalities' imaging speeds. We assessed the feasibility of using a respiration‐averaged MR (AMR) method for attenuation correction of cardiac PET data in PET/MR images. First, to demonstrate the feasibility of motion imaging with MR, we used a 3T MR system and a two‐dimensional fast spoiled gradient‐recalled echo (SPGR) sequence to obtain AMR images of a moving phantom. Then, we used the same sequence to obtain AMR images of a patient's thorax under free‐breathing conditions. MR images were converted into PET attenuation maps using a three‐class tissue segmentation method with two sets of predetermined CT numbers, one calculated from the patient‐specific (PS) CT images and the other from a reference group (RG) containing 54 patient CT datasets. The MR‐derived attenuation images were then used for attenuation correction of the cardiac PET data, which were compared to the PET data corrected with average CT (ACT) images. In the myocardium, the voxel‐by‐voxel differences and the differences in mean slice activity between the AMR‐corrected PET data and the ACT‐corrected PET data were found to be small (less than 7%). The use of AMR‐derived attenuation images in place of ACT images for attenuation correction did not affect the summed stress score. These results demonstrate the feasibility of using the proposed SPGR‐based MR imaging protocol to obtain patient AMR images and using those images for cardiac PET attenuation correction. Additional studies with more clinical data are warranted to further evaluate the method.

PACS number: 87.57.uk

## I. INTRODUCTION

The advent of hybrid positron emission tomography (PET)/magnetic resonance (MR) imaging systems has brought the potential of superior diagnostic performance over PET/CT (computed tomography) in certain applications,^1^, ^2^, ^3^, ^4^, ^5^ including cardiac imaging.^6^, ^7^, ^8^, ^9^, ^10^ However, respiratory motion can compromise the quantification of cardiac PET data using MR data. This issue has already been described for PET/CT: the respiratory motion‐induced spatial mismatch between the emission data from PET and the attenuation data estimated from CT can cause moderate to severe artifacts in up to 40% of clinical cardiac PET/CT studies.^11^, ^12^ This mismatch, which reflects the different breathing states captured by the respective imaging modalities, is largely due to the disparity in modalities' imaging speeds.^(13)^ Unlike PET data, which are averaged over several minutes, each CT slice is captured in less than 1 s. Similarly, in whole‐body PET/MR imaging, MR images for attenuation correction, unlike PET data, are usually acquired using a breath‐hold Dixon sequence, which takes about 18 s for each 21 cm bed position.^(14)^ Examples of respiration associated attenuation artifacts in clinical whole‐body PET/MR have been reported by Keller et al.^(15)^ The difference in image acquisition time suggests that artifacts caused by spatial mismatch can also occur in cardiac PET/MR imaging.

For cardiac PET/CT attenuation correction, the use of respiration‐averaged CT (ACT) images has been reported to reduce respiratory motion‐induced misalignment of PET and CT images.^13^, ^16^, ^17^ Similarly, we posit that using respiration‐averaged MR (AMR) images for attenuation correction could reduce misalignment between cardiac PET and MR data and thus reduce myocardial perfusion artifacts in PET/MR images. As a proof of concept, in the present study, we: 1) proposed a spoiled gradient‐recalled echo (SPGR)‐based MR imaging protocol for obtaining cardiac AMR images under free‐breathing conditions; 2) demonstrated the feasibility of deriving attenuation maps from AMR data; and 3) evaluated the proposed technique in a patient study.

## II. MATERIALS AND METHODS

### A. Phantom study

**Figure 1:**
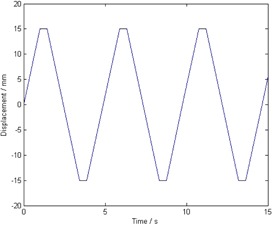
Motion trajectory of the phantom. Owing to a limitation of the motion system, a trapezoidal track was used instead of a sinusoidal one. The range (30 mm) and period (4.88 s) of the motion are reasonable parameters of a respiratory motion.

To assess the effect of respiratory motion on the proposed MR imaging protocol, we scanned a spherical phantom (diameter=16.5 cm) containing 0.1% sodium azide under simulated respiratory motion using a 3T clinical MR imaging system (GE Discovery MR750; GE Healthcare, Waukesha, WI) integrated with a motion‐enabled table (ROCKER system, GE). The spherical phantom was fixed to the top of the table. Because the table is able to generate one‐dimensional periodic motion along the axial direction of the scanner, it can be used to simulate respiratory motion, which is usually modeled as one‐dimensional motion along the superior‐inferior direction of the patient (i.e., the axial direction of the scanner). The table moves with a prescribed velocity and range; it pauses briefly at either end of the motion, leading to a trapezoidal motion track (Fig. 1). In our experiment, we used a range of ± 1.5 cm and a velocity of 1.5 cm/s, which resulted in a motion period of 4.88 s.

To obtain axial slices of the phantom under simulated respiratory motion, we performed a two‐dimensional (2D) multislice, multiphase, fast SPGR sequence (field of view=260 mm×260 mm, slice thickness=5 mm, frequency/phase encoding=128×128, repetition time [TR]/echo time [TE]=3.0 ms/1.4 ms, $\text{flip angle}=20^{\circ },\text{bandwidth}=\pm 125\;\text{kHz}$) with a single‐channel head coil. Fourteen temporal frames were acquired for each slice, and each frame's duration was 0.4 s, resulting in a temporal coverage of 5.6 s for each slice. A total of 30 slices were acquired, covering 150 mm along the axial direction. The scan duration was 169 s.

### B. Patient study

#### B.1 Scan parameters

A patient for cardiac Rubidium‐82 (^82^Rb) chloride PET/CT was recruited for the MR imaging under free‐breathing conditions. PET and MR imaging were not performed concurrently. Cardiac PET/CT was performed using a GE Discovery ST 16‐slice PET/CT system with the patient in a supine, arms‐up position. Dipyridamole stress testing with PET was performed over 6 min beginning 70 s after the start of ^82^Rb infusion (1,295–1,850 MBq [35‐50 mCi]). Dipyridamole stress testing was followed by a cine CT for ACT (8×2.5 mm X‐ray collimation, 120 kVp, 0.5 s gantry rotation, 5.9 s cine duration).^(13)^ PET perfusion images were reconstructed with filtered back projection using a Butterworth filter with a cutoff frequency of 0.55, “roll‐off” value of 10, and pixel size of $3.27\times 3.27\;{\text{mm}}^{2}$. Randoms were corrected by the option of singles, and scatter correction was applied.

For MR imaging, the patient was placed in an 8‐channel torso coil and scanned using the GE 3T MR imaging system in a supine, arms‐up position. Images of the patient's thorax and upper abdomen were obtained using the 2D multislice, multiphase SPGR sequence used for the phantom, with slightly modified parameters $(\text{TR}/\text{TE}=3.7\;\text{ms}/2.2\;\text{ms},\text{flip angle}=20^{\circ }$, frequency/phase encoding=128×128, field of view=400 mm×400 mm,slice thickness=5 mm,bandwidth=±125 kHz). In particular, TE was automatically determined by the console as the result of choosing the “in phase” setting, which ensures the phase difference between water and fat signals in the MR image is minimized. TR was automatically adjusted to account for the change in TE. Axial slices were acquired for attenuation correction of the PET images. A total of 30 slices were acquired, covering 150 mm along the superior‐inferior direction. The acquisition time for each 2D frame was 0.48 s, and 12 temporal frames (5.76 s) were obtained consecutively to ensure continuous and sufficient coverage of at least one respiratory cycle for each slice location. The temporal coverage was close to 5.9 s, the duration chosen in a previous ACT study which was based on recorded breathing cycles for 600 patients.^(13)^ The total scan duration was slightly less than 3 min, a typical PET acquisition time in oncology.

#### B.2 Data processing

Previously developed segmentation‐based methods produce attenuation maps which assign discrete attenuation coefficients for each tissue class. As a result, these methods cannot directly convert AMR images into synthetic ACT images, ${\text{ACT}}_{\text{AMR}}$, whose attenuation properties should reflect the motion blurring. Direct conversion from AMR to ${\text{ACT}}_{\text{AMR}}$ can be potentially achieved with a pattern recognition/machine learning algorithm combined with a dedicated ACT/AMR atlas,^(18)^ or fuzzy segmentation of the MR images. In the present study, we circumvented this problem by processing each MR image frame acquired at different temporal phases instead of processing AMR images. After each frame of the MR image was converted into a synthetic CT image, ${\text{ACT}}_{\text{AMR}}$ was derived as the average of all the frames for each slice.

A simple three‐class (air, lung, and soft tissue) segmentation approach^19^, ^20^, ^21^ was adopted to convert MR images into synthetic CT images. To overcome the low signal‐to‐noise ratio and spatial inhomogeneity in each frame of the MR images, we implemented the following steps to achieve better segmentation. First, anisotropic diffusion filtering^(22)^ was applied to reduce noise while preserving edge information. Then, sequential morphological erosion/dilation algorithms, which aim to remove small, isolated noise clusters that were treated as “soft tissue” during the initial thresholding, were used to threshold‐segment and refine the soft tissue. Bone voxels could not be separately identified with the obtained MR image; instead, they were incorporated into the soft tissue class during segmentation. After the soft tissues were identified, the rest of the pixels in each 2D image were grouped into connected regions using a modified Moore‐Neighbor tracing algorithm.^(23)^ The region that contained pixels outside the body contour was identified as air, while all the regions inside the body were identified as lung. After segmentation, predetermined CT numbers were assigned to each segmented class to generate a corresponding synthetic CT image. Finally, the averaged attenuation images were derived as the arithmetic mean of the individual synthetic CT images of all phases.

The assigned CT numbers for lung and tissue were determined by segmenting CT images obtained in cardiac PET/CT. Non‐anatomical components (scanner table, blanket, etc.) were first removed from the CT images, and then the lung was segmented using a region growing algorithm with a fixed upper threshold (−350 HU). Tissue was segmented by applying a lower threshold (−500 HU) and then excluding the segmented lung. Both fat and bone were included in the soft tissue class. Class‐specific mean CT numbers were then calculated from the segmented tissue classes and used to create attenuation images.

We used two sets of CT numbers to generate attenuation images from MR data. In one set, the CT numbers were from the patient who underwent MR imaging (patient‐specific [PS]). This set was created to ensure that the attenuation property of the created image matched with that of the patient. In the other set, the CT numbers were the mean of class‐specific mean CT numbers from a 54‐patient reference group (reference group [RG]). This set was created to ensure that average attenuation could also be used for attenuation correction. The average attenuation images derived using these two sets of CT numbers — ACT_AMR‐PS_ and ACT_AMR‐RG_ — were used along with the original ACT data for attenuation correction of the PET data.

Before performing attenuation correction, we removed the table in the ACT data so that the ACT data matched the AMR data. Both the ACT‐ and AMR‐derived attenuation images were manually shifted to ensure good alignment with the emission images in the myocardium region. To reduce subjectivity, two independent observers verified the results of the manual registration. Attenuation correction of the PET data was then performed with the ACT‐ and AMR‐derived attenuation images, the results of which are referred to as ${\text{PET}}_{\text{ACT}}$, PET_AMR‐PS_, and PET_AMR‐RG_, respectively.

#### B.3 Assessing differences in attenuation‐corrected PET images

Quantitative difference in the myocardium region was evaluated. The myocardium was segmented in ${\text{PET}}_{\text{ACT}}$ using a region growing algorithm with the lower threshold set at 50% of the maximal myocardium activity. We evaluated the myocardial quantification difference between MR‐based and CT‐based PET data by comparing voxel‐by‐voxel difference and mean slice activity (MSA). To assess the potential clinical impact resulting from the quantification difference, we used a semi‐quantitative five‐point scoring system^(24)^ to evaluate the reformatted 17‐segment perfusion map for each attenuation‐corrected PET dataset. The definitions of these quantities are described below.

For each voxel, the relative difference $d_{1}$ and absolute relative difference $d_{2}$ were computed as:

(1) $$\begin{array}{l} d_{1}=\frac{I-I_{REF}}{I_{REF}}\times 100\%\\ d_{2}=\frac{I-I_{REF}}{I_{REF}}\times 100\% \end{array}$$

where *I* and $I_{\text{REF}}$ represent the measured uptake in each voxel. For a slice z, the MSA was first computed as:

(2) $$MSA_{z}=\frac{\sum_{j\in M_{z}}I_{PET}(j)}{N_{z}}$$

where *j* is the index for voxel, $M_{z}$ is the set of voxels in slice z that were identified as myocardium, and $N_{z}$ is the size of $M_{z}$. For comparison, normalized mean slice activity (nMSA) was calculated as:

(3) $$nMSA_{z}=\frac{MSA_{z}}{MSA_{\text{max}}}\times 100\%$$

where ${\text{MSA}}_{\text{max}}$ is the maximal MSA of all slices in the attenuation‐corrected PET datasets. The difference in MSA in slice z was calculated as:

(4) $$dMSA_{z}=\left|nMSA_{z,AMR}-nMSA_{z,ACT}\right|$$

The polar perfusion maps were created with the Emory Cardiac Toolbox (ECToolbox, Atlanta, GA) using the 17‐segment model recommended by the American Heart Association.^(25)^ Based on the amount of perfusion present in each segment, a score ranging from 0 to 4 was assigned automatically by the software as an indicator of cardiac perfusion function (0=normal,1=equivocal,2=moderately reduced,3=severely reduced,4=absent).

## III. RESULTS

### A. AMR images of the phantom and patient

**Figure 2:**
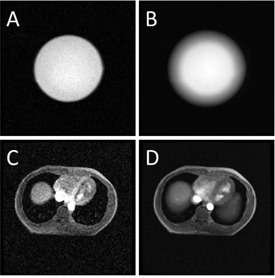
A single‐frame phantom image (a) and a corresponding motion‐averaged phantom image (b); a single‐frame patient image (c) and a respiration‐averaged patient image (d). Motion blurring is clearly visible in the averaged images.

**Figure 3:**
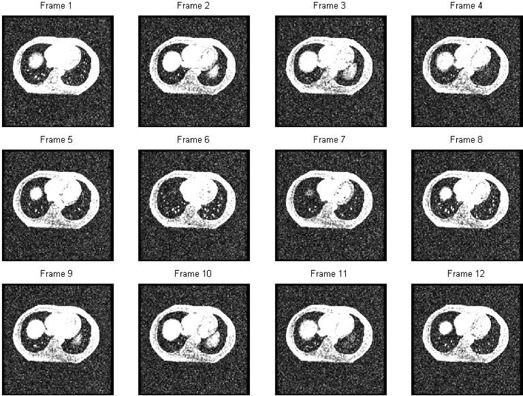
All 12 frames of one slice from the patient MR study acquired with the 2D FSPGR sequence. These frames are displayed at the signal intensity level of noise; motion ghosting artifacts that usually affect clinical MR images are not visible in these images, which were acquired under free‐breathing, indicating the effectiveness of the proposed MR protocol.

For both the phantom under simulated respiratory motion and the patient under free‐breathing conditions, visual inspection of the acquired MR images revealed that the proposed MR protocol could generate AMR images without visible motion artifacts and with average motion blurring effect, which is crucial to the success of the proposed technique (Fig. 2). For the patient study, motion artifacts were not visible in the individual frames, even when the images are displayed at the signal intensity level of noise, indicating the effectiveness of the proposed MR protocol for free‐breathing MR acquisition (Fig. 3).

### B. Class‐specific mean CT numbers

The mean CT numbers calculated for lung and tissue were −726 HU and 47 HU, respectively, for the patient and −727±51 HU and 4±18 HU, respectively, for the 54‐patient reference group. The same segmentation parameters were used for all datasets.

### C. Quantification of PET_AMR_

**Figure 4:**
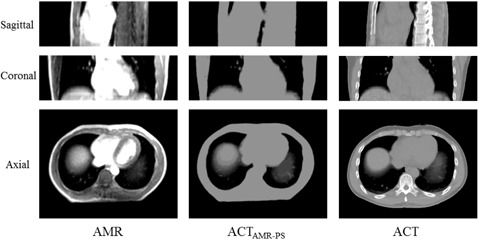
Representative slices of the sagittal, coronal, and axial views created from AMR, ACT_AMR‐PS_, and ACT data. Views created from ACT_AMR‐RG_ data, which are not visually different from those created from ACT_AMR‐PS_ data, are not shown. ACT_AMR‐PS_ and ACT images are shown under the same window setting. Respiration averaging effects in the AMR images were preserved in the MR‐derived attenuation images. Bones were included in the soft tissue class. Spatial mismatch between AMR and ACT images, which was the result of different table shapes, is clearly present in the dorsal area.

Representative slices of the sagittal, coronal, and axial views created from AMR, ACT_AMR‐PS_, and ACT data are shown in Fig. 4. With ${\text{PET}}_{\text{ACT}}$ as the reference, the differences $d_{1}$ and $d_{2}$ of PET_AMR‐PS_ were −2.0%±5.1% and 4.3%±3.3%, respectively; for PET_AMR‐RG_, the differences were −6.2%±5.0% and 6.3%±4.8%, respectively.

**Figure 5:**
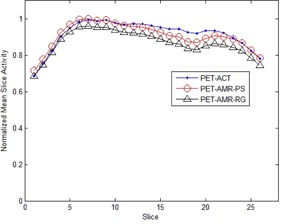
Mean myocardial uptake in different slices normalized to maximal mean uptake. The slices are labeled from the most superior slice to the most inferior slice. The three curves were from PET corrections with ACT, AMR‐PS (patient specific) and AMR‐RG (reference group).

The nMSA at different PET_AMR‐PS_, PET_AMR‐RG_, and ${\text{PET}}_{\text{ACT}}$ slices are plotted in Fig. 5. The highest MSA value was that of slice 7 of PET_AMR‐PS_, and this value was used to normalize all three datasets. The absolute quantification difference in mean myocardial activity between PET_AMR‐PS_ and ${\text{PET}}_{\text{ACT}}$ at different slices was 2.0%±1.6%; the maximum difference was 5.0%. The absolute quantification difference between PET_AMR‐RG_ and ${\text{PET}}_{\text{ACT}}$ was 4.7%±2.5%, with a maximum difference of 8.8%.

**Figure 6:**
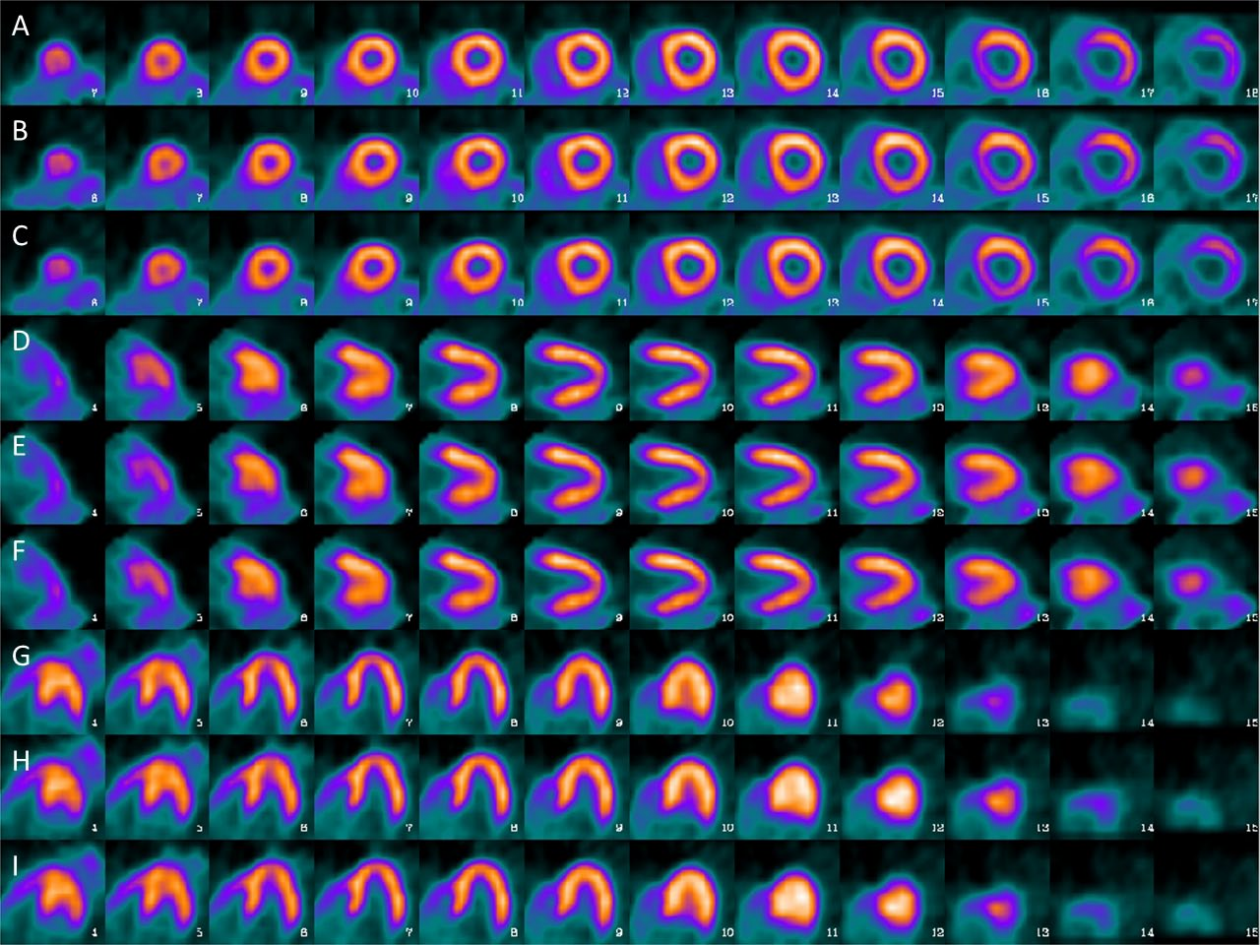
Reformatted myocardial PET images showing different cardiac axes, in which rows A–C are short‐axis views, rows D–F are horizontal long‐axis views, and rows G–I are vertical long‐axis views. In each three‐row section, ${\text{PET}}_{\text{ACT}}$, PET_AMR‐PS_, and PET_AMR‐RG_ images appear in sequence from top to bottom.

**Figure 7:**
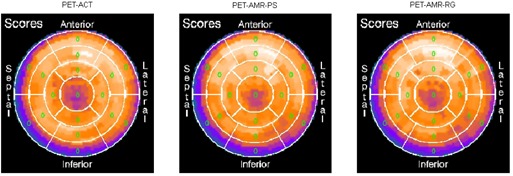
PET_ACT_, PET_AMR‐PS_, and PET_AMR‐RG_ myocardial perfusion images. Stress scores were assigned to each segment. The patient had normal cardiac function, with a summed stress score of 0 in the ${\text{PET}}_{\text{ACT}}$ image. Using MR‐derived attenuation images for attenuation correction did not affect the stress scores.

Reformatted PET images (PET_ACT_, PET_AMR‐PS_, and PET_AMR‐RG_ images) of the myocardium, along the short axis, horizontal long axis, and vertical long axis, are shown in Fig. 6. The PET images of the left ventricle in the attenuation‐corrected PET images were reformatted into the polar maps using a 17‐segment model. In the original ACT‐corrected PET image, the summed stress score was 0, indicating normal cardiac function. The scores in all segments were the same in both AMR‐corrected PET datasets (Fig. 7).

## IV. DISCUSSION

The different patient tables of the MR system and the PET/CT system caused a visible spatial mismatch between AMR and ACT images in the dorsal area of the patient (Fig. 4). (The MR system's table has a flat surface, whereas the PET/CT system's table has a curved surface.) This mismatch likely contributed to the underestimation of the cardiac activity in PET_AMR‐PS_ and PET_AMR‐RG_, which would not be an issue in a combined PET/MR system. In the present study, we reduced the impact of this spatial mismatch on PET quantification by ensuring the alignment of the attenuation images and emission images in the myocardium region. This was achieved with manual registration, a typical approach to correct misregistration between cardiac PET and CT images.^(12)^

In a previous phantom‐based study, Zhang et al.^(26)^ found that reconstructed PET activity can be underestimated by 10%–20% if one does not correct the attenuation the MR system's table contributes. Our phantom experiment, using the PET/CT system with its curved table, produced similar results. Therefore, the imaging system table must be considered to accurately quantify PET data. In the present study, we attempted to incorporate the PET/CT system's table into the AMR‐derived attenuation images. Unfortunately, this proved to be difficult due to the mismatch of the patient's body contour resulting from the table difference. For the purpose of fair comparison, therefore, we removed the table from the ACT images before performing the attenuation correction.

As a proof of concept, we used a three‐class segmentation scheme to derive MR‐based attenuation images. Despite its simplicity, this method achieved relatively accurate quantification in the reconstructed PET images. While creating the attenuation map from AMR images, we did not perform bone segmentation, which is difficult without the aid of a dedicated ultrashort echo time imaging sequence and, to date, has been mainly applied in PET/MR imaging of the brain.^27^, ^28^ In brain PET, ignoring bone has been suggested to cause quantification bias.^(29)^ In whole‐body PET/MR imaging, however, neglecting bone in segmented attenuation images has been suggested to cause large errors only in regions that are inside or near bones.^30^, ^31^, ^32^ In one example demonstrated by Samarin et al.,^(32)^ classifying bone as soft tissue resulted in less than 6% difference for PET voxels in the heart region. Ouyang et al.^(33)^ also concluded that three‐class segmentation can be sufficient for PET quantification in the heart, as it yields less than 5% quantification difference after compensation. These studies indicate that bone segmentation may not be necessary for cardiac PET/MR.

Martinez‐Moller et al.^(14)^ proposed using a four‐class segmentation scheme with the Dixon technique, in which fat is separated from nonfat soft tissue and assigned a different attenuation coefficient. Although evaluating different segmentation‐based attenuation correction methods was beyond the scope of our study, it should be noted that the Dixon technique can be integrated into our proposed AMR protocol with a modification of the MR sequence, to separate fat and nonfat soft tissue while maintaining similar temporal resolution. Such an approach may improve PET quantification in patients with higher body fat composition.

To investigate the impact of assigned CT numbers on quantification, we created two sets of attenuation images from MR data: ACT_AMR‐PS_ and ACT_AMR‐RG_. As expected, ACT_AMR‐PS_ resulted in a smaller quantification difference, owing to the more accurately estimated attenuation coefficients for the patient. In clinical PET/MR applications, however, patient‐specific CT images are usually unavailable, and general coefficients must be used for attenuation correction. In the present study, the mean lung CT number of the patient (−726 HU) was close to that of the reference group (‐727±51 HU); however, a Student's *t*‐test revealed that the patient's mean tissue CT number was significantly higher than that of the reference group (47 HU vs. 4±18 HU, p<0.001). As a result, the quantification difference in the ACT_AMR‐RG_‐corrected PET data (6.3%) was higher than that in the ACT_AMR‐PS_‐corrected PET data (4.3%); however, the error was small. For patients whose mean attenuation coefficients or CT numbers deviate less from the population mean, less quantification difference is expected. AMR‐based attenuation correction did not affect the summed stress score, indicating that the quantification difference is not clinically significant in this one case. Further investigation is required to evaluate the clinical impact of the proposed method of MR‐based attenuation correction.

Several authors have proposed MR‐based respiratory motion correction for thorax PET/MR,^34^, ^35^, ^36^, ^37^ and at least one phantom‐based study tested a tagged MR imaging‐based technique for cardiac motion correction.^(38)^ Although such approaches aim to eliminate the impact of motion in the reconstructed PET image, they usually require nonstandard MR sequences that are not clinically available. In contrast, the approach we propose uses a scheme that has been proven effective in PET/CT to reduce the spatial mismatch between emission and attenuation data and the consequent artifact in the cardiac perfusion PET image. The present study's findings suggest that a similar improvement can be achieved in cardiac PET/MR imaging without resorting to motion correction.

In the present study, we tested the feasibility of using AMR images of the thorax to create attenuation maps for cardiac PET data. As a proof of concept, we designed a simple strategy to include the motion blurring effect by processing the images of individual phases. This strategy does not fully capture the motion blurring effect and is a potential limitation of this study. However, the quantification errors were small, suggesting that this simple strategy is feasible. In future work we will investigate methods that directly convert AMR into motion‐blurred attenuation images.

## V. CONCLUSIONS

The present study's findings demonstrate the feasibility of using AMR images for attenuation correction of cardiac PET data. Despite the fact that the different tables of the MR and PET/CT systems caused a geometrical mismatch of the AMR‐based and ACT‐based attenuation images, the PET data corrected with the MR images achieved accurate quantification and maintained the same summed stress score. Further study with more patients is warranted to determine the effectiveness of AMR‐based attenuation correction in cardiac PET/MR imaging.
